# Collective Dynamics of Neural Networks With Sleep-Related Biological Drives in Drosophila

**DOI:** 10.3389/fncom.2021.616193

**Published:** 2021-05-03

**Authors:** Shuihan Qiu, Kaijia Sun, Zengru Di

**Affiliations:** ^1^International Academic Center of Complex Systems, Beijing Normal University at Zhuhai, Beijing, China; ^2^School of Systems Science, Beijing Normal University, Beijing, China

**Keywords:** coupled neural network, LFP, network structure, synchronization, duration of sleep and wake

## Abstract

The collective electrophysiological dynamics of the brain as a result of sleep-related biological drives in Drosophila are investigated in this paper. Based on the Huber-Braun thermoreceptor model, the conductance-based neurons model is extended to a coupled neural network to analyze the local field potential (LFP). The LFP is calculated by using two different metrics: the mean value and the distance-dependent LFP. The distribution of neurons around the electrodes is assumed to have a circular or grid distribution on a two-dimensional plane. Regardless of which method is used, qualitatively similar results are obtained that are roughly consistent with the experimental data. During wake, the LFP has an irregular or a regular spike. However, the LFP becomes regular bursting during sleep. To further analyze the results, wavelet analysis and raster plots are used to examine how the LFP frequencies changed. The synchronization of neurons under different network structures is also studied. The results demonstrate that there are obvious oscillations at approximately 8 Hz during sleep that are absent during wake. Different time series of the LFP can be obtained under different network structures and the density of the network will also affect the magnitude of the potential. As the number of coupled neurons increases, the neural network becomes easier to synchronize, but the sleep and wake time described by the LFP spectrogram do not change. Moreover, the parameters that affect the durations of sleep and wake are analyzed.

## 1. Introduction

Recently, the collective dynamics of the brain has become a very hot topic because of its wide applications in sleeping, associative memories, image processing, learning, disease, and so on. In particular, synchronization is one of the most important collective dynamics of neural networks and plays an important role in brain activity. Many research results on synchronization have been extensively reported (Mirollo and Strogatz, 1990; Maex and Schutter, 2003; Hammond et al., 2007; Buzsaki and Watson, 2012; Noah et al., 2018; Muhammet et al., 2019). In Muhammet et al. (2019), the authors researched the reason for the spontaneous termination phenomenon of neurons, and three different coupling methods, i.e., gap junctions, and the excitatory or inhibitory synapses of neurons, were considered. Noah et al. (2018) investigated the synchronization dynamics and spiking patterns of thalamic neurons and gave the membrane voltage of thalamic neurons to show the process of the brain from sleep to wake. Therefore, it is a critical step to understand how neural systems work in the brain. In particular, some phenomena are related to sleep.

We spend one third of our life asleep, however, how the brain changes during sleep and wake is still not clear. Thus, research on brain sleep is important and valuable (Hendricks et al., 2000; Shaw et al., 2000; Tononi and Cirelli, 2003; Bushey et al., 2011; Donlea et al., 2011; Xie et al., 2013; Dissel et al., 2015; Watson and Buzsaki, 2015; Liang et al., 2017; Melvyn et al., 2017). In Melvyn et al. (2017), observed the activity of the brain of Drosophila and applied several experimental methods, such as heating, the use of gaboxadol, genetic activation, and so on, to induce sleep. The brain activity of Drosophila during induced and spontaneous sleep was compared by performing local field potential recordings. The alternation of slow wave sleep epochs and rapid-eye movement sleep is important for sleep. These dynamic sleep processes were deemed to be unique to birds, initially because rapid-eye movement sleep is helpful for animals that have the ability to close or move their eyes. However, some analogous sleep function memory consolidation exists in Drosophila (Shaw et al., 2000; Donlea et al., 2011). Dissel et al. (2015) studied some behaviors of Drosophila mutants after sleep and discovered that sleep induction can improve learning. However, the abovementioned references on fly sleep are all experimental studies. In Liang et al. (2017) studied the Drosophila circadian neural circuit using whole-brain imaging *in vivo*. This is an experimentally-validated model of interactions among circadian neurons to translate the phase of the molecular clock into neuronal activity. As a matter of fact, mathematical modeling has become an important tool for understanding the dynamics of neural networks (Smolen et al., 2002; Fathallah-Shaykh et al., 2009; Noah et al., 2018; Jin et al., 2019). Noah et al. constructed a minimal model of four coupled conductance-based neurons to study spiking patterns and synchronization dynamics of thalamic neurons along the sleep-wake cycle in Noah et al. (2018). Jin et al. (2019) introduced electromagnetic induction and its noise in the model and investigated their effects on the regulation of sleep wake cycle. In Smolen et al. (2002), the authors reduced a previously detailed model to a minimal representation of the transcriptional regulation essential for circadian rhythmicity in Drosophila. Unfortunately, very few research results on the study of the collective dynamics of fly brain during sleep by constructing a neural network model have been published. Thus, constructing a neural network model for fly sleep would be beneficial.

The local field potential (LFP) refers to the low-frequency part (usually less than 500 Hz) of the extracellular voltage signal recorded in the brain and can record the activity of many neurons near the electrodes. Thus, LFP is helpful and useful for researching the dynamics of local networks, such as cognitive processes including memory, attention and perception (Colgin et al., 2009), sensory processing (Montemurro et al., 2009), etc. A large number of papers on fly sleep also made use of LFP from sleeping flies to analyze their brain activity (van Swinderen et al., 2004; van Alphen et al., 2013; Melvyn et al., 2017; Troup et al., 2018). The authors found that LFP activity is reduced during spontaneous sleep in flies but is increased when sleep is induced. Moreover, 7–10 Hz oscillations can be observed via spectrograms of the LFP in both spontaneous and induced sleep (Melvyn et al., 2017). Similarly, the sleep in flies is related to decreased LFP activity (van Swinderen et al., 2004; van Alphen et al., 2013). Hence, the LFP is a powerful tool for analyzing the collective dynamics of brain neurons. On the other hand, the network structure is also important for researching the LFP. Different network structures may lead to different results in many situations.

In this study, the collective electrophysiological dynamics of coupled neurons in the fly brain via sleep-related biological drives was considered. First, the conductance-based neurons were extended to a coupled neural network to simulate sleep neuron activity in the fly brain. Then, two different metrics (mean value and dependence of the single-neuron distance) were used to estimate the LFP. Based on the two different metrics, qualitatively similar results were obtained that were roughly consistent with the experimental results. Third, the effects of different network structures on the LFP were examined, and these structures were divided into three cases: (1) a grid connection, (2) a random increase in the number of long-range connections of each neuron by 5, 20, and 50 based on the grid connection, and (3) the Watts-Strogatz (WS) small world networks. These results indicate that different LFP time series can be obtained under different network structures and the density of the network will also affect the magnitude of the potential, and the suitable network structure should develop significantly low average distance while maintaining its large clustering coefficient. As the number of coupled neurons increases, the network becomes synchronized, but no impact on the duration of sleep and wake is described by the LFP spectrogram. Finally, the influence of parameters related to the coupling strength and time constant on the duration of sleep and wake was considered. The results show that the coupling strength *g*_*gj*_ has no effect on the duration of sleep and wake, however, the duration of sleep and wake are positively correlated with *τ*_1_, *τ*_2_.

## 2. Models and methods

A single neuron of our networks is modeled based on the Huber-Braun thermoreceptor model (Braun et al., 2003) as follows:

$$C\frac{dV_{i}}{dt}=-I_{l_{i}}-\alpha (I_{Na_{i}}+I_{K_{i}})-\beta (I_{pNa_{i}}+I_{K,Ca_{i}})-I_{gj_{i}}-I_{ext_{i}}$$

where *i* = 1, 2, ⋯ , *n* represents the number of neurons; *V*_*i*_ is the membrane potential of the *i*-th neuron; *C* is the membrane capacitance; *I*_*l*_*i*__ is the leakage current of the *i*-th neuron:

(1) $$I_{l_{i}}=g_{l_{i}}(V_{i}-E_{l})$$

where *g*_*l*_*i*__ is the conductance and *E*_*l*_ is the equilibrium potential. *I*_*Na*_*i*__ and *I*_*K*_*i*__ are the fast depolarizing and repolarizing currents for the spike generation of the *i*-th neuron, respectively; *I*_*pNa*_*i*__ and *I*_*K,Ca*_*i*__ are slow currents for subthreshold oscillations; And the role of the parameters *α*, *β* is to alter the magnitude of the spike currents or subthreshold currents separately. The voltage-dependent currents are given in the following equations:

(2) $$\begin{array}{l} I_{j_{i}}=g_{j_{i}}a_{j_{i}}\left(V_{i}-V_{j}\right) \end{array}$$

where *j* = *Na, K, pNa, K, Ca*.

(3) $$\begin{array}{l} a_{Na_{i}}=\frac{1}{1+exp\left[-s_{Na}\left(V_{i}-V_{0Na}\right)\right]} \end{array}$$

(4) $$\begin{array}{l} \tau_{K}\frac{da_{K_{i}}}{dt}=\frac{1}{1+exp\left[-s_{K}\left(V_{i}-V_{0K}\right)\right]}-a_{K_{i}} \end{array}$$

(5) $$\begin{array}{l} \tau_{pNa}\frac{da_{pNa_{i}}}{dt}=\frac{1}{1+exp\left[-s_{pNa}\left(V_{i}-V_{0pNa}\right)\right]}-a_{pNa_{i}} \end{array}$$

(6) $$\begin{array}{l} \tau_{K,Ca}\frac{da_{K,Ca_{i}}}{dt}=-\eta I_{pNa_{i}}-ka_{K,Ca_{i}} \end{array}$$

where *τ*_*j*_ is the time delay; *s*_*j*_ is the slope of the activation curve; *V*_0*j*_ is the half-activation potential; *η* is the coupling constant; and *k* is the relaxation factor. *I*_*gj*_*i*__ is the total synaptic current received by neurons *c*_1_, *c*_2_, ⋯ , *c*_*i*_. The model's parameter values are listed in Table 1 and the parameter values are very similar as proposed in an earlier preliminary study (Svetllana et al., 2011). For a gap junction, the synaptic current is

(7) $$\begin{array}{l} I_{gj_{i}}=\underset{k\in neighbors\left(i\right)}{\sum }g_{gj}\left(V_{k}-V_{i}\right) \end{array}$$

where neighbors (*i*) is the set of neighbors of neuron *i*; *g*_*gj*_ is the coupling strength; And *I*_*ext*_*i*__ is the external input of the *i*-th neuron from sleep-related biological drives used to regulate the sleep-wake cycles. The sleep-related biological drives include interconnected positive and negative feedback loops (Smolen et al., 2002; Fathallah-Shaykh et al., 2009; Liang et al., 2017). In this paper, a simplified model that represents the dynamics of the positive and negative feedback loops of the Drosophila oscillator was used (Smolen et al., 2002). A negative feedback loop is included, in which PER protein represses *per* transcription by binding the dCLOCK transcription factor. A positive feedback loop is also included, in which dCLOCK indirectly enhances its own formation.

(8) $$\begin{array}{l} I_{ext_{i}}=I_{dclock_{freei}}=I_{dclocki}-I_{peri} \end{array}$$

(9) $$\begin{array}{l} I_{dclocki}=g_{dclocki}\left[dCLOCK\right]\left(E_{syn}-V_{i}\right) \end{array}$$

(10) $$\begin{array}{l} I_{peri}=g_{peri}\left[PER\right]\left(E_{syn}-V_{i}\right) \end{array}$$

The differential equations for [dCLOCK] and [PER] are based on an earlier published model of the Drosophila circadian oscillator (Smolen et al., 2002).

(11) $$\begin{array}{l} \frac{d\left[dCLOCK\right]}{dt}=v_{sc}R_{sc}-k_{dc}\left[dCLOCK\right] \end{array}$$

(12) $$\begin{array}{l} R_{sc}=<\frac{K_{2}}{K_{2}+\left[dCLOCK_{free}\right]}>\tau_{2} \end{array}$$

(13) $$\begin{array}{l} \frac{d\left[PER\right]}{dt}=v_{sp}R_{sp}-k_{dp}\left[PER\right] \end{array}$$

(14) $$\begin{array}{l} R_{sp}=<\frac{\left[dCLOCK_{free}\right]}{K_{1}+\left[dCLOCK_{free}\right]}>\tau_{1} \end{array}$$

where [*dCLOCK*_*free*_] = [*dCLOCK*] − [*PER*] or zero, whichever is greater. *τ*_1_ denotes the time delay between *per* transcription and the synthesis of new PER protein. *τ*_2_ means the time delay between *dclock* transcription and the synthesis of new dCLOCK protein. The models (11)–(14) have been described in detail before (Lema et al., 2000; Smolen et al., 2001, 2002) so here we only provide a brief summary of the unified model and report model parameters in Table 1 for completeness.

**Table 1:** The parameters values for the model.

**Parameter**	**Value**	**Unit**	**Parameter**	**Value**	**Unit**
C	1	*μF*/*cm*^2^	*g*_*l*_	0.4	*mS*/*cm*^2^
*V*_*l*_	–60	*mV*	*g*_*Na*_	1.3	*mS*/*cm*^2^
*V*_*Na*_ = *V*_*syn*_	50	*mV*	*g*_*K*_	1.75	*mS*/*cm*^2^
*V*_*k*_	–90	*mV*	*g*_*pNa*_	0.22	*mS*/*cm*^2^
*V*_0*Na*_ = *V*_0*K*_	–25	*mV*	*g*_*K,Ca*_	0.35	*mS*/*cm*^2^
*V*_0*pNa*_	–40	*mV*	*g*_*gj*_	0.0001	*mS*/*cm*^2^
*s*_*Na*_ = *s*_*K*_	0.25	*mV*^−1^	*τ*_1_	10	*h*
*s*_*pNa*_	0.09	*mV*^−1^	*τ*_2_	10	*h*
*η*	0.012	*cm*^2^/*μA*	*v*_*sp*_	0.5	*nMh*^−1^
*k*	0.17	⋯	*v*_*sc*_	0.25	*nMh*^−1^
*τ*_*K*_	0.000875	*s*	*k*_*dp*_	0.5	*h*^−1^
*τ*_*pNa*_	0.00425	*s*	*k*_*dc*_	0.5	*h*^−1^
*τ*_*K,Ca*_	0.00875	*s*	*K*_1_	0.3	*nM*
*α* = *β*	4	⋯	*K*_2_	0.1	*nM*
*g*_*dclock*_ = *g*_*per*_	0.05	*mS*/*cm*^2^	*E*_*syn*_	50	*mV*

*The parameter values are very similar as proposed in an earlier preliminary study (Smolen et al., 2002; Svetllana et al., 2011)*.

**Figure 1:**
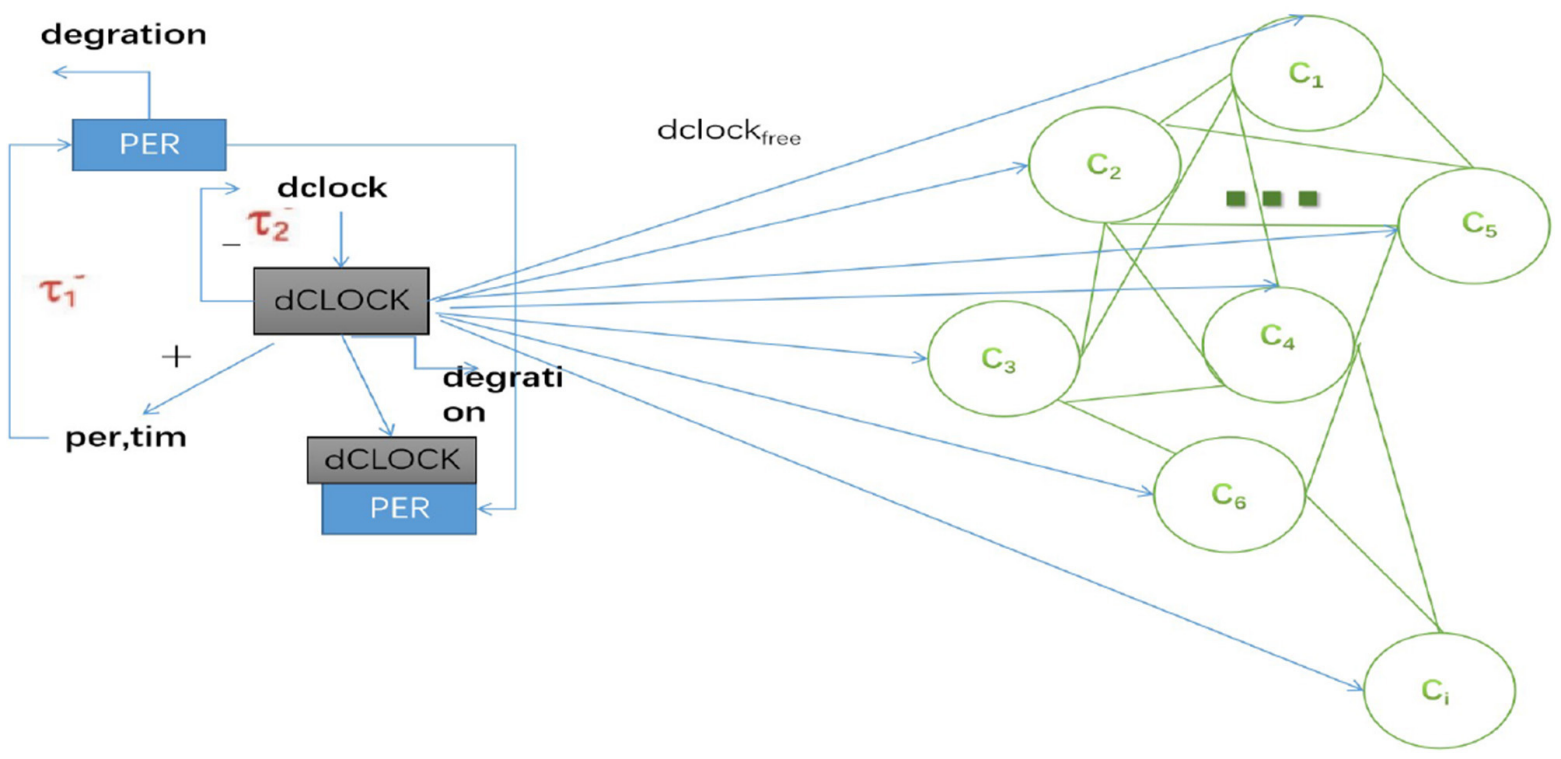
Schematic of the coupled neuron by sleep-related biological drives in Drosophila.

In this paper, we established a coupled neuron network for studying collective electrophysiological dynamics of Drosophila during sleep and wake. In the network model, many neurons (*C*_1_, *C*_2_, ⋯ , *C*_*i*_) are coupled via gap junctions, and all receive an excitatory input from sleep-related drives, as shown in Figure 1. The sleep-related drives have one positive and negative feedback loop. dCLOCK activates *per* transcription and thus PER synthesis. PER represses *per* transcription (and thus PER synthesis) by binding dCLOCK. PER also activates dCLOCK synthesis by binding dCLOCK and relieving dCLOCK's repression of dclock transcription. During wake, neurons (*C*_1_, *C*_2_, ⋯ , *C*_*i*_) receives circadian current input *I*_*dclock*_*free*__ and dCLOCK activates *per* to synthesize PER and the current *I*_*dclock*_*free*__ = 0 during sleep.

To observe the activity of the Drosophila brain during sleep and wake, we make observations after 24 h to ensure the validity of the results.

To better understand the brain activity during wake and sleep, it is important to research the LFP of the Drosophila brain. In this paper, the LFP is estimated by two methods. One is to approximate the LFP with the average value of the membrane potential of the whole network:

$$\Phi =\frac{\sum_{i=1}^{n}V_{i}}{n}$$

and the other is to consider the distance-dependent LFP (Lindn et al., 2011). All neurons are distributed around the electrode on a two-dimensional plane. We calculate the distance *r*_*i*_ of neurons from the electrode by the distance between two points and then add up the LFPs of each neuron to obtain the LFP of the entire network.

(15) $$\begin{array}{l} \Phi =\overset{n}{\underset{i=1}{\sum }}V_{i}f\left(r_{i}\right) \end{array}$$

(16) $$\Phi =\sum_{i=1}^{n}V_{i}f(r_{i})\;f(r_{i})=\{\begin{array}{cc} 1 & r_{i}<\theta \\ \theta^{\gamma }r_{i}^{-\gamma } & r_{i}\geq \theta \end{array}$$

where *r*_*i*_ is the distance from the *i*-th neuron to the electrode; *f*(*r*_*i*_) is the single-neuron shape function; *γ* ≥ 0 is a decay exponent; And *θ* is the cutoff distance to avoid a singularity.

## 3. Results

To understand the dynamics of the brain by the sleep-related biological drives in Drosophila, we first research the dynamics of neurons with a fully coupled network in section 3.1. Then, we explore the influence of the network structures on the LFP in section 3.2. Finally, we investigate the effects of different parameters on Drosophila sleep in section 3.3.

### 3.1. Dynamics of Neurons in Drosophila Brain

**Figure 2:**
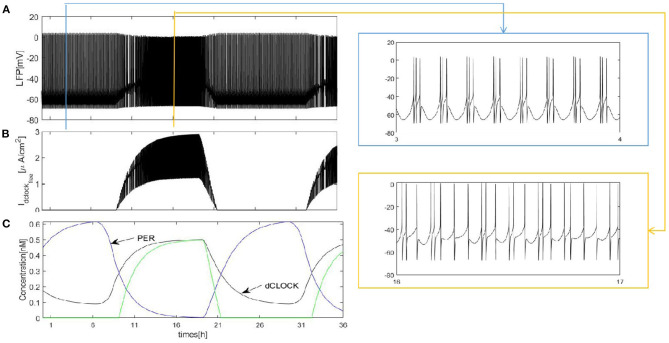
Dynamics of coupled neurons in Drosophila brain over 24 h cycles. **(A)** The LFP is calculated by the mean value. The network size *N* = 100 and the mean connectivity z = 99. The coupling strength ${\text{g}}_{\text{gj}}=0.0001\text{mS}/\text{c}{\text{m}}^{2}$. **(B)** I_dclock_free__ represent postsynaptic current from the dCLOCK. **(C)** Simulation of circadian oscillations. Blue(PER), black(dCLOCK), green(dCLOCK_free_).

We first construct a fully coupled network with size *N* = 100. How the spiking patterns of coupled neurons are changed by means of sleep-related biological drives is investigated first. The results are shown in Figure 2. The overall oscillation activity of the entire network during sleep and wake is reflected by the LFP signal in Figure 2A (calculated by the mean value). During wake, due to PER protein by binding the dCLOCK transcription factor represses per transcription, the protein enhances its own formation and the postsynaptic current increases. The LFP exhibits irregular and regular spike firing and has a regular spike toward the end of the wake episode (Figure 2A, orange box). During sleep, PER protein starts to accumulate by dCLOCK activates per transcription and the current I_dclock_free__ = 0. This leads to a transition to regular bursting in coupled neurons (Figure 2A, blue box). In Figure 2C, the circadian oscillator has a positive and a negative feedback loops (Smolen et al., 2002). During sleep, dCLOCK (Figure 2C, black) starts to decrease because it need to activate per transcription to synthesize PER and thus PER protein is getting more and more (Figure 2C, blue). During wake, the PER protein binds dCLOCK and thereby represses per transcription, and activates dCLOCK synthesis. dCLOCK begins to become more and more and decreased concentration of PER. According to Figure 2C, the input current is zero during sleep and increases during wake in Figure 2B.

**Figure 3:**
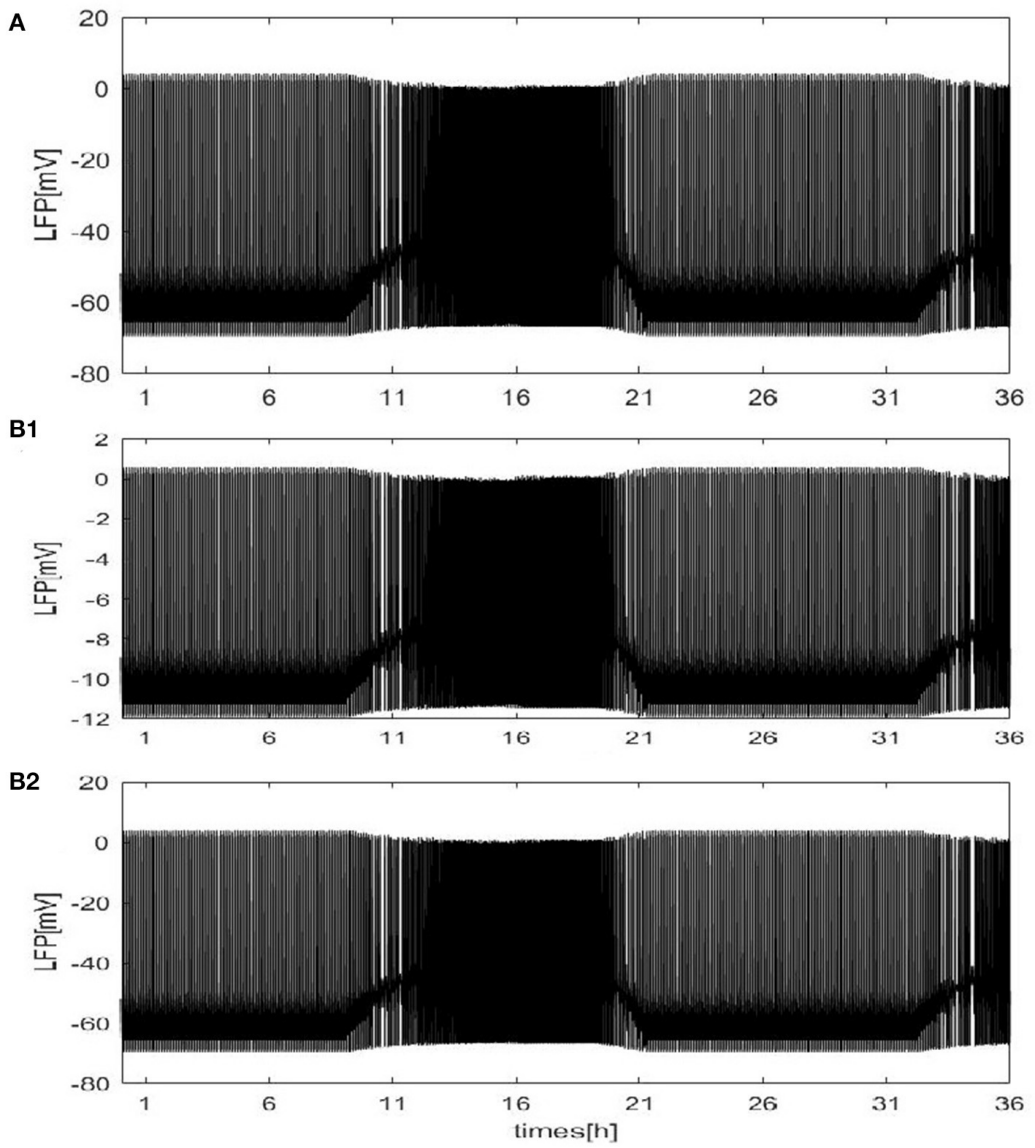
The LFP of coupled neurons in Drosophila brain over 24 h cycles. The LFP is calculated by using the dependence of the single-neuron distance. The network size *N* = 100 and the mean connectivity z = 99. The decay exponent *γ* = 2 and cutoff distance *θ* = 10 *μm*. The coupling strength ${\text{g}}_{\text{gj}}=0.0001\text{mS}/\text{c}{\text{m}}^{2}$. **(A)** The distribution of neurons is a circle. The radius is 100*μ*m and the position of the electrode at the center of the circle. **(B)** The distribution of neurons is a grid distribution. (**B_1_**) The coordinate of the first neuron is C_1_(100, 100) in the grid distribution and the position of the detection point at (150, 500), (**B_2_**) the position of the detection point is changed to (200, 500).

Except for the above results, the LFP can be estimated with our neural network mode by using the dependence of the single-neuron distance. The network size *N* = 100, and the mean connectivity z = 99. Twodifferent distributions are considered: a circle distribution (Figure 3A) and a grid distribution (Figure 3B). For the grid distribution, 100 neurons (*C*_1_, *C*_2_, ⋯ , *C*_100_) are divided into a 10 × 10 square matrix in the coordinate system, and the distance between each neuron and its neighboring neurons is 100 *μm*. These results are similar to Figure 2A. The difference is shown for the grid distribution in Figure 3b_1_. The overall LFP decreased. But, the result will change if the position of the detection point is changed. For example, if the position of the detection point changed from (150, 500) to (200, 500) (Figure 3b_2_), the overall LFP becomes almost the same as in Figure 2A. Thus, Based on methods of mean value and the distance-dependent LFP, qualitatively similar results were obtained.

### 3.2. The Network Structure Effect for LFP

The LFP is one of the experimental measures of neural activity and has wide application. Recently, many research results of experiments investigating Drosophila sleep were reported by using the LFP. To understand the network structure effect, we examine the LFP of a coupled neural network under different network structures in this subsection.

**Figure 4:**
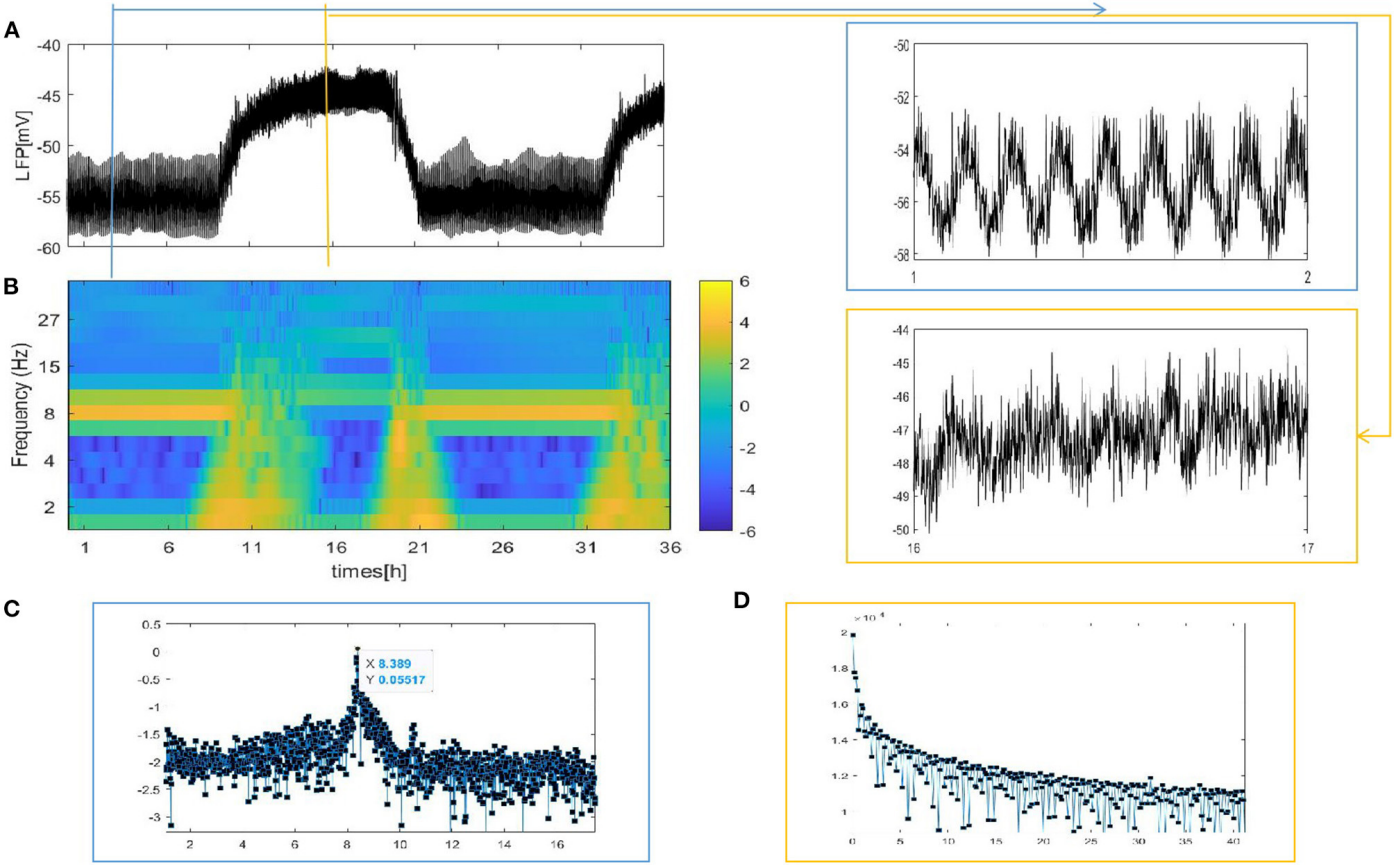
The LFP signal and spectrogram of the coupled neuron in grid network (z = 4). **(A)** The full and detailed graphs of LFP are given, sleep (blue box), wake (orange box). **(B)** Spectrogram of 2–40 Hz LFP power are shown during sleep and wake. **(C,D)** Power spectra for sleep (blue box) and wake (orange box).

To test the importance of the network structure, we observe the LFP signal and spectrogram of the LFP for a fly recording over 24 h. We discuss that the neurons in the network are partially coupled. Specifically, the partial coupling is divided into three cases. In the first case, each neuron is coupled to the upper, lower, left, and right neuron (grid connection). Based on the first case, each neuron is randomly increased by 5, 20, and 50 long-range connections. The last case involves the WS small world networks and the mean connectivity z = 4, 24, 54, respectively by random rewiring of three percent of links of a regular ring (p = 0.03). (Note that in each case, except for the different network structures, the other parameters are the same. The initial value is random. The LFP signal diagram and spectrogram are simulated by using the mean value.) The first structure that we consider is the grid connection. The network size *N* = 100, and the mean connectivity z = 4. The results are shown in Figure 4.

Different from the fully coupled network in Figure 2A, i.e., the LFP amplitude decreases during sleep compared to that during wake. Actually, many researchers have reported that sleep in Drosophila is associated with, on average, decreased LFP activity compared to wake (Nitz et al., 2002; van Swinderen et al., 2004; van Alphen et al., 2013). The corresponding spectrogram of the LFP is shown in Figure 4B. We find obvious 7–10 Hz dominated oscillations during sleep that were absent during wake. For further confirmation, we apply the Fourier transform to the LFP (Figures 4C,D). An oscillation at approximately 8 Hz appears in sleeping flies (blue box) but was absent in awake flies (orange box). These results are roughly consistent with the experimental data shown in Figures 1C,E in the research article by Melvyn et al. (2017).

**Figure 5:**
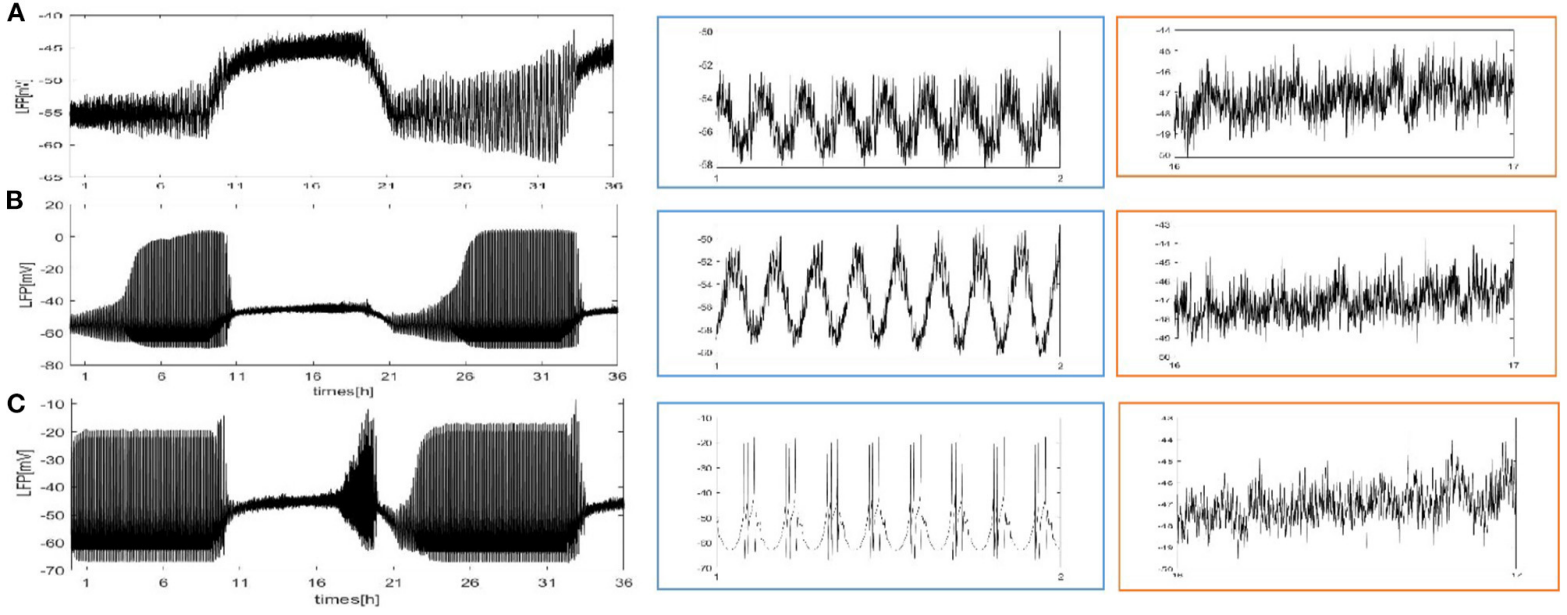
The full and detailed LFP signal of each neuron in the network randomly adds 5, 20, and 50 long-range connections. **(A)** Each neuron is randomly increased by 5 long-range connections (z = 9). **(B)** Each neuron is randomly increased by 20 long-range connections (z = 24). **(C)** Each neuron is randomly increased by 50 long-range connections (z = 54).

**Figure 6:**
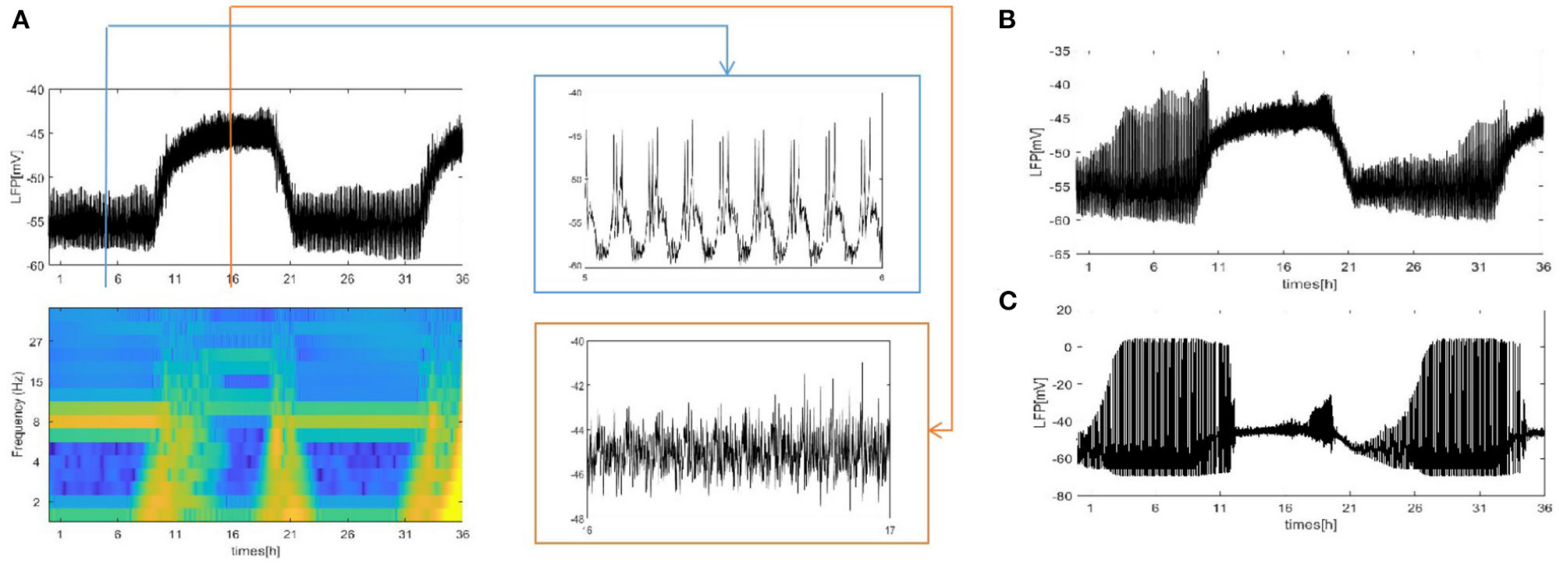
The LFP of the coupled neuron in WS small world network by random rewiring of three percent of links of a regular ring (*p* = 0.03). **(A)** The LFP signal and spectrogram of WS small world network with the mean connectivity z = 4. Sleep (blue box), wake (orange box). **(B)** The LFP signal of WS small world network with the mean connectivity z = 24. **(C)** z = 54.

We also consider the case where each neuron is randomly increased by 5, 20, and 50 long-range connections. The obtained results are shown in Figure 5. Obviously, the LFP signal changes under different network structures. During sleep and wake, the spike pattern of LFP remains almost unchanged see blue and orange box. During sleep, the oscillation are all regular bursting and it is just that the LFP becomes more ambiguous when the number of coupled neuron is not enough. The oscillation are all irregular or chaos spikes during wake. Moreover, the full LFP signals are different under different network structures. In Figure 5B, compared with Figure 5A, the LFP decreases first and then increases during sleep, and all decreases during wake. In Figure 5C, during sleep, the LFP becomes similar to LFP of neurons are fully coupled in Figure 2A, however, the LFP still decreases during wake. Obviously, these network structures (Figures 5B,C) that increases the degree of nodes in the network on average are unreasonable. The corresponding spectrogram is not much different from that of previous results (not shown). The last structure that we consider is the WS small world network with the mean connectivity z = 4, 24, 54 by random rewiring of three percent of links of a regular ring (p = 0.03) in Figure 6. The results are similar to those of the evolution process of Figure 4. It is worth noting that the network structure (Figure 6C) also is unreasonable due to the decreased LFP activity during wake. We try to change the connection probability *p* and find the LFP will change, for example, the LFP of WS small world network with the mean connectivity *z* = 24 when *p* = 0.5 is similar to Figure 6C (not show). Moreover, as the connection probability *p* is lowered, and the LFP of WS small world network with the mean connectivity *z* = 54 will not change. Thus, we concluded that the suitable network structure should develop significantly low average distance while maintaining its large clustering coefficient.

**Figure 7:**
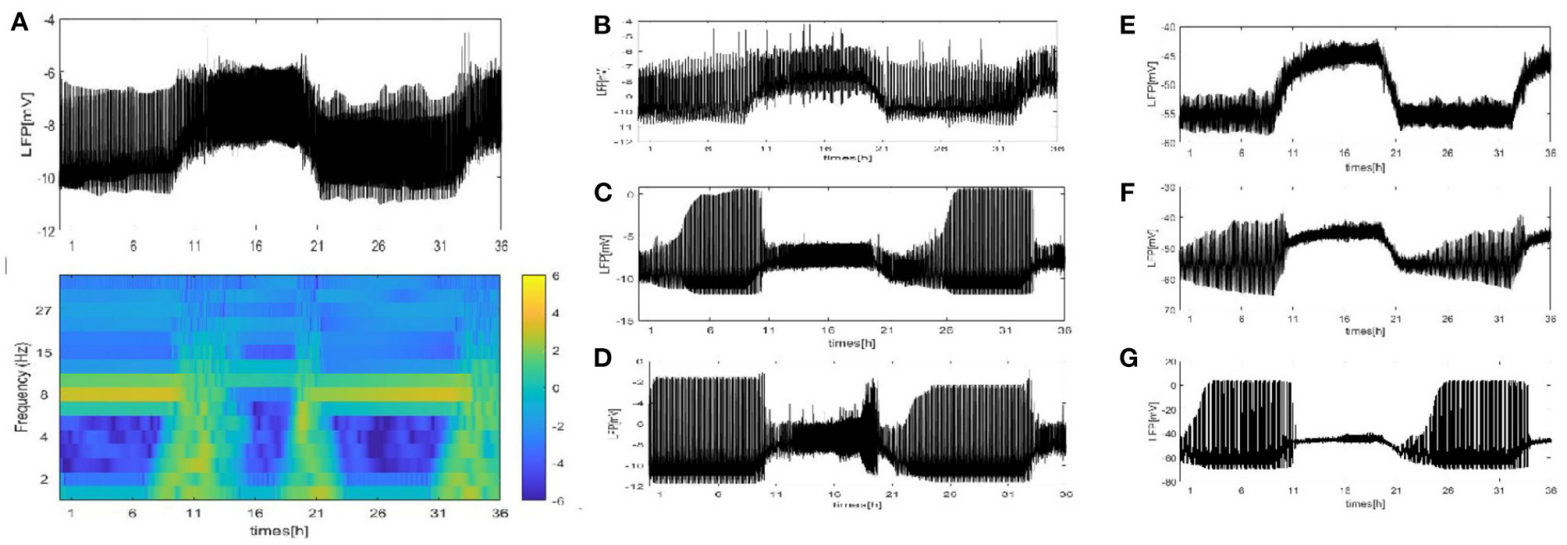
The LFP by using dependence of the single-neuron distance under seven different network structure. **(A)** The signal and spectrogram of LFP are shown at (z = 4). **(B–D)** Each neuron is randomly increased by 5, 20, and 50 long-range connections (z = 9, 24, 54). **(E–G)** WS small world networks with the mean connectivity z = 4, 24, 54 by random rewiring of three percent of links of a regular ring (*p* = 0.03).

We perform a similar analysis of the case where the LFP is calculated by using the dependence of the single-neuron distance in Figure 7. (Note that we consider that all neurons are distributed around the electrode on a two-dimensional plane. There exists two distributions: the grid distribution and the circle distribution. We calculate the distance of neurons from the electrode as the distance between two points, and for the grid distribution in Figures 7A–D, we assume that the position of the electrode is (150, 500) and that of the first neuron *C*_1_ is (100, 100). For the circle distribution in Figures 7E–G, the radius is 100 and the position of the electrode at the center of the circle.) All results on the LFP under the seven different network structures are shown in Figure 7. Particularly, the LFP and the corresponding spectrogram of the grid distribution at z = 4 are given in Figure 7A. Compared with the mean value method, the difference is that the LFP power is decreased. The other results are similar to those in Figures 4–6. These results indicate that regardless of which method is used, qualitatively similar results are obtained. Furthermore, the time series of the LFP is sensitive to the underlying structure, but the underlying structure has almost no influence on the spiking patterns and spectrum.

**Figure 8:**
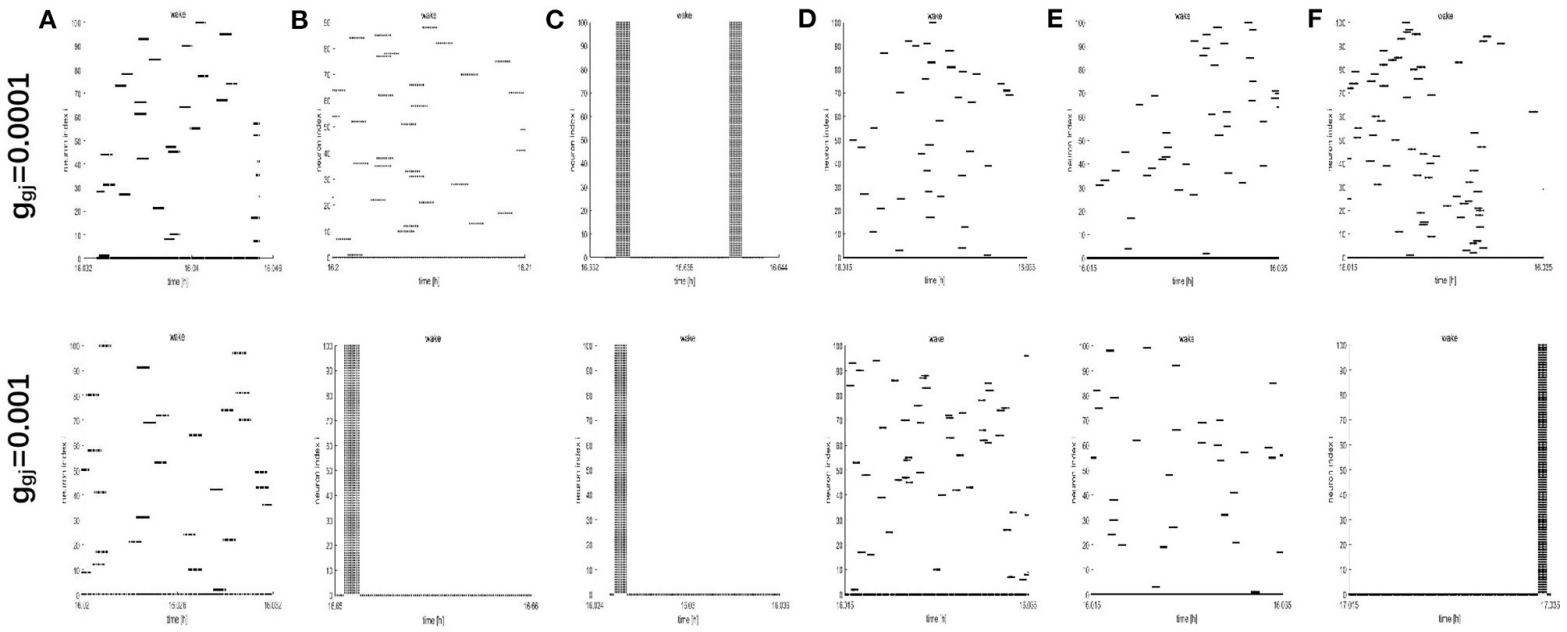
The raster plots for different network structures and coupling strength during wake. **(A–C)** The grid connection and each neuron is randomly increased by 20 and 50 long-range connections (z = 4, 24, 54). **(D–F)** The WS small world networks with mean connectivity z = 4, 24, 54 by random rewiring of three percent of links of a regular ring (*p* = 0.03).

**Figure 9:**
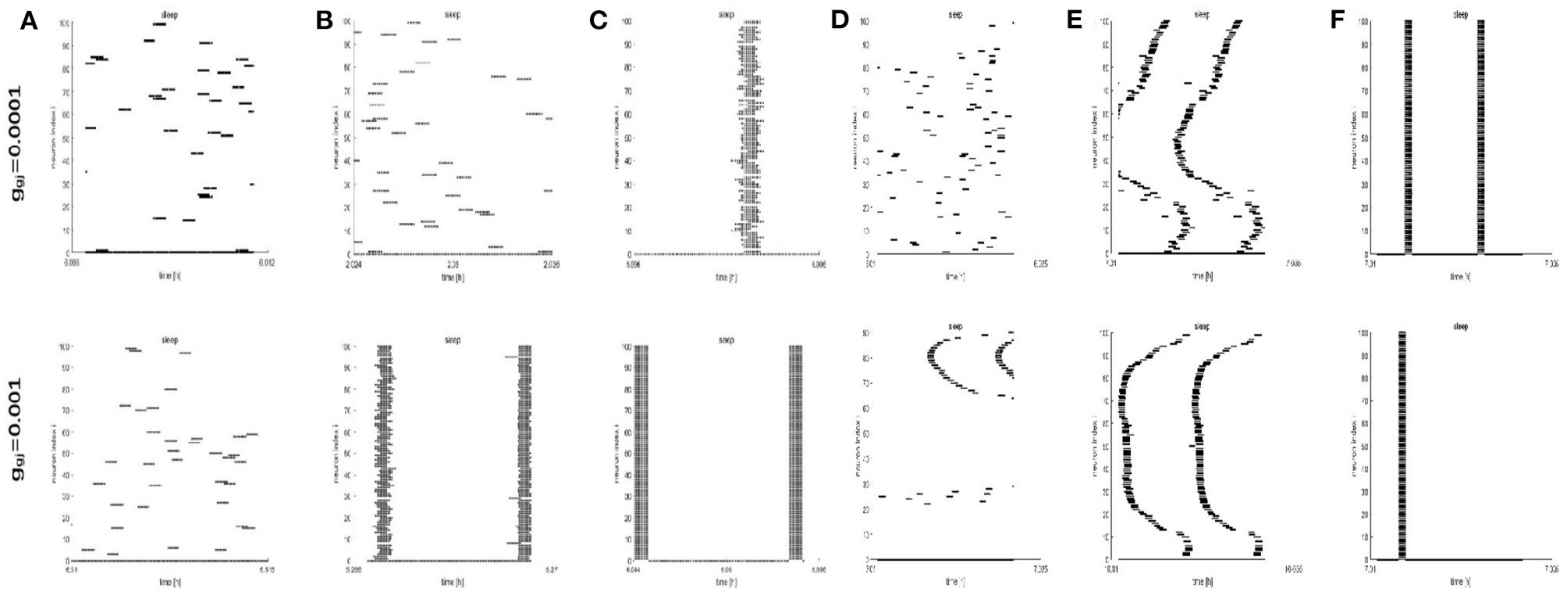
The raster plots for different network structures and coupling strength during sleep. **(A–C)** The grid connection and each neuron is randomly increased by 20 and 50 long-range connections (z = 4, 24, 54). **(D–F)** The WS small world networks with mean connectivity z = 4, 24, 54 by random rewiring of three percent of links of a regular ring (*p* = 0.03).

To further study the dynamics of neurons, we investigated the raster plots for different network structures. These raster plots for different network structures are shown in Figures 8, 9 (a-c: regular networks and add long-range connections. d-f: WS small world networks). The results in Figure 8 show that the synchronization of neurons during wake when the coupling strength is 0.0001 and 0.001, respectively. During wake, as the number of coupled neurons in a network increases, the synchronization of coupled neurons is obvious (Figures 8A–C) except for the WS small world networks (Figures 8D–F, top). And especially, we noticed that as an increasing number of neurons are coupled with each other, the coupling strength has a great influence on the synchronization of neurons. The network (z = 24, Figure 8B) and the WS small world network (z = 54, Figure 8F) are almost completely synchronized by comparing the upper figures When *g*_*gj*_ = 0.001. Therefore, we concluded that the synchronization is not only related to the network structures but also to the coupling strength. During sleep, the corresponding results are shown in Figure 9. The obtained results for the synchronization of neurons in regular network (Figures 9A–C) are similar to those results during wake. Interestingly, the synchronization transition is accompanied with a hysteresis loop for the WS small world networks (Figures 9D–F), and this type of synchronization is different from the regular networks (Figures 9A–C). This interesting result may indicate the earlier published that the brain networks at the microscopic level are similar to WS small world networks (Shih et al., 2015; Scheffer and Meinertzhagen, 2019).

### 3.3. Effects of Parameters on the Collective Dynamics

The sleep time of flies can be changed by, e.g., heating, and drugs, in an experimental environment. Therefore, we need to investigate the sleep and wake time of flies by adjusting the parameters. Based on the discussion in section 3.2, we conclude that different network structures and methods for estimating the LFP have an impact on LFP signals but hardly affect the spiking patterns. Therefore, for the rest of this paper, we report the results of calculating the LFP based only on the mean value, and for the network structure, each neuron randomly adds 5 long-range connections (*z* = 9). (Note that to validate the results, the initial value is random. We changed only the parameters of our research; the other parameters remain unchanged. For each parameter of our research, we repeat the experiment ten times to calculate the LFP amplitudes in each frequency band and sleep time. The final results are obtained after taking the average.)

**Figure 10:**
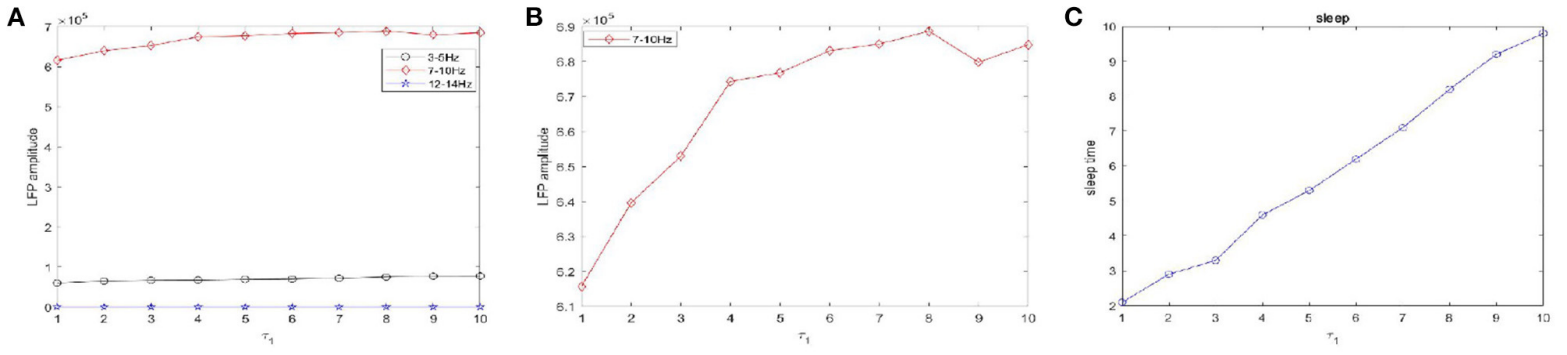
The amplitudes of LFP and sleep time change under different values of the time constant τ_1_ during sleep. **(A)** The amplitude of the LFP in the three different frequency bands were calculated respectively, 3–5 Hz (black), 7–10 Hz (red), 12–15 Hz (blue). **(B)** The amplitude of the LFP in 7–10 Hz (red). **(C)** The changes of the sleep time.

**Figure 11:**
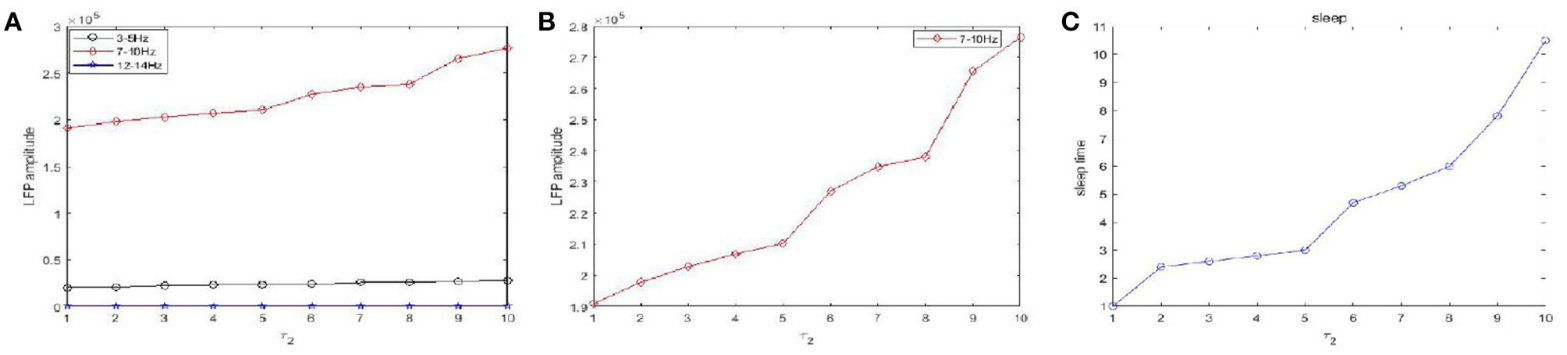
The amplitudes of LFP and sleep time change under different values of the time constant *τ*_2_ during sleep. **(A)** The amplitude of the LFP in the three different frequency bands were calculated respectively, 3–5 Hz (black), 7–10 Hz (red), 12–15 Hz (blue). **(B)** The amplitude of the LFP in 7–10 Hz (red). **(C)** The changes of the sleep time.

**Figure 12:**
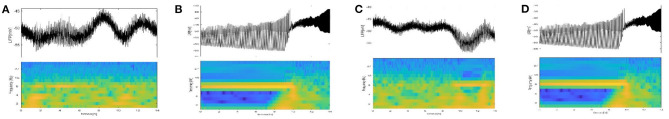
The LFP oscillation and corresponding spectrograms at *τ*_1_, *τ*_2_. **(A)** *τ*_1_ = 1 h. **(B)** *τ*_1_ = 10 h. **(C)** *τ*_2_ = 1 h. **(D)** *τ*_2_ = 10 h.

We consider the network coupling strength *g*_*gj*_ and time constant *τ*_1_, *τ*_2_. *τ*_1_ denotes the time delay between per transcription and the synthesis of new PER protein and *τ*_2_ denotes the time delay between dclock transcription and the synthesis of new dCLOCK protein (Smolen et al., 2002). These results are given in Figures 10–**12**. The amplitudes of the LFP changes under different coupling strengths during sleep (not shown). We find clear amplitudes that are very large in the 7–10 Hz frequency bands during sleep and in other frequency bands that are not obvious. These observations show that the oscillations are mainly concentrated in the 7–10 Hz range. As the coupling strength increases, the amplitudes of the LFP remain almost unchanged. The sleep time under different coupling strengths is kept at approximately 10 s. Therefore, the sleep and wake time of flies cannot be changed by increasing the coupling strength.

Figure 10 shows the amplitudes of the LFP (Figures 10A,B) and sleep time change (Figure 10C) under different values of the time constant *τ*_1_ during sleep. The amplitudes are very high in the 7–10 Hz (red) frequency bands and are not obvious in other frequency bands. Obviously, as the time constant *τ*_1_ increases, overall, the amplitudes of the LFP (red) are increased. We also observe that the sleep time increases creases with increasing time constant *τ*_1_. Moreover, the amplitude of the LFP (Figure 10B) does not vary monotonically with increasing *τ*_1_ but reveals a fluctuating behavior. Based on the different values of the time constant *τ*_2_, the changes in the amplitudes of the LFP and sleep time are displayed in Figure 11. Similar to the results in Figure 10, overall, the amplitudes of the LFP (Figure 11B) are increased. Meanwhile, *τ*_2_ also has a positive effect on the sleep time (Figure 11C).

For further verification, a diagram of the LFP oscillation and the corresponding spectrograms at *τ*_1_ = 1*h*, 10*h* (Figures 12A,B) and *τ*_2_ = 1*h*, 10*h* (Figures 12C,D) are given. The time of LFP bursting is the same as the duration of the frequency band around 8 HZ. The time of bursting is significantly longer in Figure 12B and the frequency band around 8 HZ is significantly longer in Figure 12D.

## 4. Conclusion

In this work, we studied the collective dynamics of the brain in Drosophila by sleep-related biological drives. Four coupled conductance-based neurons were extended to coupled neural networks to simulate sleep neurons activity in the fly brain. The neurons are coupled by electrical gap junctions and adjusted via sleep-related biological drives. The sleep-related biological drives include interconnected positive and negative feedback loops. A negative feedback loop is included, in which PER protein represses per transcription by binding the dCLOCK transcription factor. A positive feedback loop is also included, in which dCLOCK indirectly enhances its own formation.

To understand the difference between sleep and wake in flies, we used two methods to estimate the LFP by means of the given neural network. Regardless of the method, the results show that the LFP signal becomes regular bursting, and a 7–10 Hz oscillation appears on the spectrogram of the LFP during sleep. The LFP displays the chaos state (irregular and regular spikes that occasionally burst) distributed in different frequency bands during wake. Therefore, based on the two different methods, qualitatively similar results, roughly consistent with the experimental results, obtained.

To further study this phenomenon, the effect of the network structure is considered. First, we use the mean value method to estimate the LFP. Partial coupling is considered in the network, involving (1) grid connections, (2) a random increase in the number of long-range connections for each neuron by 5, 20, and 50, and (3) WS small world networks. The acquired results for the time series of an LFP are different under different network structures, as shown in Figures 4–6. Actually, many researchers have reported that sleep in Drosophila is associated with, on average, decreased LFP activity compared to wake. Therefore, we concluded that the suitable network structure should develop significantly low average distance while maintaining its large clustering coefficient and the duration of sleep and wake does not change as the network structures change. Then, the synchronization of neurons is considered by raster plots. The raster plots show that the neurons become easier to synchronize as the number of coupled neurons increases and that also synchronization becomes more obvious as the coupling strength increases. However, there exists an interesting result that the synchronization transition is accompanied with a hysteresis loop for the WS small world networks (see Figures 9D–F), and this type of synchronization is different from the regular networks (Figures 9A–C). This interesting result may indicate the earlier published that the brain networks at the microscopic level are similar to WS small world networks. Moreover, the dependence of the single-neuron distance is used to calculate the LFP. Under different network structures, all results are similar to the results of the mean value in addition to the amplitude of the LFP. Thus, these results indicate that the suitable network structure should develop significantly low average distance while maintaining its large clustering coefficient, and as the number of coupled neurons increases, the network becomes synchronized, but no impact on the duration of sleep and wake is described by the spectrogram of the LFP.

Many papers on induced sleep in flies have been reported. The sleep time of flies can be changed by heating, drug injection, etc., in an experiment. Similarly, we can check whether several parameters in a network affect the sleep time. We first consider the coupling strength *g*_*gj*_, the results of which show that the amplitude of the LFP remains almost unchanged when the coupling strength *g*_*gj*_ is increased and has no effect on the sleep time. Then, the time constants *τ*_1_, *τ*_2_ are examined. The results are different from those of the coupling strength *g*_*gj*_. The time constants have a large impact on the sleep time because *τ*_1_ denotes per transcription and the synthesis of new PER protein and *τ*_2_ denotes the time delay between dclock transcription and the synthesis of new dCLOCK protein. The sleep time is positively correlated with the time constant *τ*_1_, *τ*_2_.

This work preliminarily simulated the areas related to sleep in the Drosophila brain and can be extended to the study of the underlying sleep mechanism. In this paper, we considered only sleep neurons coupled by gap junctions. In future research, these neurons may include combinations of excitatory and inhibitory synapses and be controlled by other neurons. The sleep-related drives can also be extended. In practice, there are many neurons in the brain that implement complex functions. Therefore, sleep-related drives can included as other related drivers to make the network more complete and more practical. Moreover, the impact of environmental interference cannot be ignored. It is more practical and valuable to add environmental interference to the model and observe the collective dynamics of the brain in Drosophila.

## Data Availability Statement

The original contributions presented in the study are included in the article/supplementary material, further inquiries can be directed to the corresponding author/s.

## Author Contributions

ZD: design and conduct of the study. SQ and KS: preparation, review, or approval of the manuscript. All authors contributed to the article and approved the submitted version.

## Conflict of Interest

The authors declare that the research was conducted in the absence of any commercial or financial relationships that could be construed as a potential conflict of interest.
